# Mannan-Rich Fraction Supplementation: A Promising Nutritional Strategy for Optimizing Growth and Health of Pre-Weaning Calves

**DOI:** 10.3390/ani15121684

**Published:** 2025-06-06

**Authors:** Shanshan Guo, Yanfei Feng, Jianhao Yang, Haomiao Zhao, Jiajun Ma, Yuan Zhang, Mengkun Sun, Yifan Li, Gang Lin, Pengfei Lin, Aihua Wang, Yaping Jin

**Affiliations:** 1 College of Veterinary Medicine, Northwest A&F University, Yangling, Xianyang 712100, China; 13155556229@163.com (S.G.); 17837798963@163.com (Y.F.); 15836581038@163.com (J.Y.); 15670597653@163.com (H.Z.); 2023055530@nwafu.edu.cn (Y.Z.); 15667200523@163.com (M.S.); yifanli18931178421@163.com (Y.L.); linpengfei@nwsuaf.edu.cn (P.L.); aihuawang1966@163.com (A.W.); 2 Key Laboratory of Animal Biotechnology, Ministry of Agriculture and Rural Affairs, Northwest A&F University, Yangling, Xianyang 712100, China; 3 Ningxia Xingyuanda Agriculture and Animal Husbandry Co., Ltd., Lingwu 750406, China; 13259563331@163.com; 4 Key Laboratory of Agrifood Safety and Quality, Institute of Quality Standards and Testing Technology for Agricultural Products, Chinese Academy of Agricultural Sciences, Ministry of Agriculture and Rural Affairs, Beijing 100081, China; ganglin.cau@gmail.com

**Keywords:** MRF, calves, growth performance, gut health, immunity

## Abstract

The early-stage feeding of calves significantly impacts their lifelong productive performance. During this period, the calves’ digestive tracts are in a dynamic state of development, making nutritional regulation a key factor in optimizing their growth potential. This research investigated the influence of mannan-rich fraction (MRF) supplementation on pre-weaning calves. Sixty healthy Holstein calves were randomly divided into a control group and three MRF-supplemented groups (seven males and eight females per group) with low (L-MRF, 2.5 g/calf per day), medium (M-MRF, 5 g/calf per day), and high (H-MRF, 10 g/calf per day) doses. Growth performance, serum biochemical indicators, organ indices, intestinal morphology, and short-chain fatty acid (SCFA) concentrations were measured over a 42-day experiment. Compared with the control group, the M-MRF group had 4.9% higher overall body weight, body length, and starter intake, alongside a reduced diarrhea incidence by 4.6%. At day 14, MRF-supplemented groups exhibited significantly lower Interleukin-6 (IL-6) and tumor necrosis factor-α (TNF-α) levels, along with significantly higher Interleukin-12 (IL-12), growth hormone (GH), and Immunoglobulin G (IgG) levels. By day 42, IL-6 and Interleukin-1β (IL-1β) levels were also significantly decreased. Additionally, the lung and liver indices were higher and villus height was greater in both the jejunum and colon with MRF supplementation. In terms of SCFAs, the acetate level in the jejunum and the butyrate level in the colon were significantly higher in the MRF-supplemented calves. Both acetate in the jejunum and butyrate in the colon showed a significantly positive correlation with growth performance. Taken together, these findings suggest that MRF supplementation (particularly at 5 g/day) supports calf growth, gut health, and immunity, making it a promising nutritional strategy for pre-weaning calves.

## Simple Summary

This study assessed the effects of dietary mannan-rich fraction (MRF, a purified form of mannan oligosaccharides) supplementation on pre-weaning calves. Calves given a medium MRF dose (5 g/calf/day) performed best: They had a 6.5% higher body weight gain and a 35.8% lower diarrhea incidence than the control group. Moreover, MRF supplementation improved lung and liver development (as seen from higher organ indices), enhanced jejunal and colonic villus height (indicating better intestinal morphology), and promoted the production of beneficial short-chain fatty acids (SCFAs) in these intestinal parts, which suggests improved gut health and nutrient absorption.

## 1. Introduction

The health and growth performance as calves marks the starting and crucial phase of a dairy cow’s life cycle, exerting a direct and long-lasting influence on the overall productivity and profitability of a dairy farm [1,2]. Calves, particularly those in the pre-weaning stage, are at a critical period of growth and development. During this time, they face numerous challenges that can affect their health and productivity, and one of the key factors influencing calf performance is nutrition [3,4,5]. The pre-weaning period is characterized by a transition from a liquid diet to solid feed. This change is not only a simple shift in food type but also poses multiple challenges to the calves’ immature digestive systems, which can be stressful for calves. This stress can lead to reduced feed intake, poor nutrient absorption, and increased susceptibility to diseases [6,7]. Moreover, the immature gut of pre-weaning calves is more vulnerable to infections and inflammation, which can further impair their growth and development [8]. Therefore, it is essential to find effective strategies to support the health and performance of calves during this critical stage.

Dietary additives such as mannan oligosaccharides (MOS), derived from yeast cell walls, have gained attention for their prebiotic properties, which include pathogen-binding capacity and stimulation of beneficial gut microbiota [9]. The Mannan-rich fraction (MRF) is a purified preparation of MOS characterized by α-(1,2)- and α-(1,3)-linked D-mannose branches attached to elongated α-(1,6)-linked D-mannose backbone structures [10]. Experimental evidence across multiple species indicates that mannan oligosaccharides (MOS) exert species-specific probiotic effects through distinct physiological pathways. Murine models reveal that dietary MOS supplementation modulates gut microbial ecology by selectively enriching saccharolytic microbiota, thereby stimulating short-chain fatty acid (SCFA) biosynthesis [11]. In the context of poultry, numerous studies have highlighted the positive impacts of MOS. Research on broilers has shown that MOS affected broiler intestinal morphology resulting in greater crypt depth and villus height [12]. This morphological change enhances nutrient absorption and overall growth. For aged laying hens, dietary MOS supplementation boosts production performance and feed conversion efficiency by improving ileal nutrient digestibility and reducing pathogenic gut bacteria [13]. In swine, adding MOS to the diet has been discovered to increase duodenal villi height, reduce mRNA levels of Tumor Necrosis Factor α and Toll-Like Receptor 4 [14]. Further research has indicated that MOS can reduce the incidence of post-weaning diarrhea by upregulating tight junction proteins’ expression and enhancing intestinal immune function [15]. In ruminants, despite their complex digestive systems, MOS demonstrates potential benefits: Supplementing MOS to dry cows enhances anti-rotavirus immunity via lymphocyte proliferation, improving colostral antibody transfer to offspring [16]. In calves, MRF and live yeast supplementation increases withers height and hip width [17], while diets containing *L. acidophilus* and MOS improve growth performance, elevate serum total protein/globulin levels, and promote beneficial gut microbiota [18]. MOS may also enhance health by increasing antibody production [19] or modulating intestinal morphology/function [20]. However, compared with poultry and swine, the application of MOS in ruminants, especially pre-weaning calves, is still in the exploratory stage, and the optimal dosage of MOS for pre-weaning calves has yet to be determined.

This study bridges the translational gap by systematically evaluating MRF (a form of MOS) supplementation in pre-weaning calves, generating comprehensive data on its effects on growth, health, and immune markers during critical early life stages. We hypothesized that 5 and 10 g/day of MRF supplementation would enhance growth performance, improve intestinal morphology, and modulate inflammatory cytokine profiles in pre-weaning calves, with 5 g/day being the optimal dosage for balancing efficacy and cost, thereby providing valuable insights for improving calf rearing practices.

## 2. Materials and Methods

The protocols and procedures used in this study were approved by Animal Ethical and Welfare Committee of Northwest A&F University (Ethical Approval number: DY2022093).

### 2.1. Experiment Design and Animal Feeding

A total of 60 healthy Holstein calves (32 females and 28 males) at 2 ± 1 d of age and body weight (BW) of 39 ± 3 kg for females and 43 ± 3 kg for males (mean ± standard deviation) were randomly assigned to 4 groups: a control group (CON) and three MRF (Alltech, Inc., Nicholasville, KY, USA)-supplemented groups. The MRF-supplemented groups received diets with low (L-MRF, 2.5 g/calf per day), medium (M-MRF, 5 g/calf per day), and high (H-MRF, 10 g/calf per day) levels of MRF, respectively. MRF treatment was mixed into the morning milk feeding. All calves received 4 L of good quality colostrum, tested by a colostrometer (Brix > 22), within 2 h after birth. Following a serum total protein (TP) assay, only calves with a TP concentration > 5.5 g/dL at 2 d of age were enrolled in the subsequent experiment. Calves were separated from their dams immediately after birth and individually housed in outdoor plastic hutches bedded with wheat straw. Prior to calf placement, each hutch was thoroughly disinfected. The calf hutches were situated in a well-ventilated area, with each hutch equipped with a pellet feeder and water bucket to ensure easy access. The calves remained in their pens for the duration of the study. This experiment lasted 42 d. Pasteurized normal milk (average milk protein and fat contents 3.44% and 3.97%, respectively) was fed twice a day at 0700 h and 1500 h from d 1 to 5 at 3.5 L/meal, from d 6 to 10 at 4 L/meal, from d 11 to 15 at 4.5 L/meal, and from d 16 to 59 at 5 L/meal. On the first day of the experiment, starter (Table 1) and water were provided for ad libitum consumption. Starter orts were collected and weighed daily.

### 2.2. Growth Measurements

The calves were weighed before afternoon feeding on days 7, 14, 21, 28, and 42 to estimate the body weight (BW) and average daily gain (ADG). The amount of starter offered and orts were recorded daily to determine dry matter intake (DMI). Total dry matter intake (Total DMI) was calculated as the sum of the DMI of milk and starter. Feed efficiency (FE) was calculated following the following formula: FE = ADG/Total DMI.

Fecal consistency scores were recorded daily for each calf throughout the trial based on a scale of 1 to 4 described by a previous study [21], where 1 represented normal, 2 indicated pasty, 3 indicated semi-liquid, and 4 indicated liquid with an abnormal color. Diarrhea was defined as a score greater than 2. The incidence of diarrhea among the calves was analyzed every week using the following formula [10]:

Diarrhea rate = (the number of calves with diarrhea × the number of days with diarrhea)/(total number of calves in each group × the experimental days) × 100%.(1)

### 2.3. Serum Biochemical Indicators

On days 14 and 42, before the morning feeding, 5 mL of blood was collected from the jugular vein of the 10 calves (5 males and 5 females) in each group using blood collection tubes without additives. The blood samples were handled gently to avoid hemolysis and were kept in a tilted position until serum separation occurred at room temperature. Subsequently, the samples were centrifuged at 3000× *g* for 15 min at 4 °C to isolate serum, which was then aliquoted into 1.5 mL tubes and stored at −20 °C until analysis. The levels of Interleukin-2 (IL-2), Interleukin-6 (IL-6), Interleukin-10 (IL-10), Interleukin-12 (IL-12), Interleukin-1β (IL-1β), Tumor Necrosis Factor-α (TNF-α), growth hormone (GH), Immunoglobulin A (IgA), Immunoglobulin G (IgG), and Immunoglobulin M (IgM) were analyzed using ELISA kits (Enzyme-linked Biotechnology Co., Ltd., Shanghai, China) following the manufacturer’s instructions.

### 2.4. Slaughter and Organ Index Measurement

On day 42, 6 male calves from each control group and M-MRF group were randomly selected and slaughtered following the approved ethical guidelines. Following slaughter, the abdominal cavity was promptly opened, and the gastrointestinal tract was extracted. The organs (lung, heart, liver, and spleen) were carefully dissected out and gently rinsed with pre-cooled physiological saline to remove surface blood and debris and were then blotted dry with absorbent paper. The wet weight of each organ was immediately measured using an analytical balance and recorded. The organ index was calculated according to the following formula:

Organ Index (%) = Organ Wet Weight(g)/Body Weight(g) × 100%(2)

In addition, the digesta from the jejunum and colon were removed, and the tissues were rinsed with a 0.9% saline solution. Tissue samples (2 cm × 2 cm) from the central region of the small intestine were preserved in 4% formaldehyde and then embedded in paraffin blocks, sectioned, and stained for morphological analysis Furthermore, portions of the jejunum and colon tissues were kept at −80 °C for subsequent detection.

### 2.5. Biochemical and Immunological Parameters in Jejunum and Colon

The levels of sIgA (secretory immunoglobulin A), IL-2, IL-6, IL-10, IL-12, IL-1β, and TNF-α in the jejunal and colonic tissue were measured using ELISA kits (Enzyme-linked Biotechnology Co., Ltd., Shanghai, China) according to the manufacturer’s instructions.

### 2.6. SCFA Concentrations

The concentrations of SCFAs in the intestinal digesta were determined using gas chromatography (Trace-1310) following the procedure detailed by the previous study [22].

### 2.7. Statistical Analysis

Statistical analyses were performed using SPSS 27.0 (IBM, Armonk, NY, USA). Periodically collected variables (BW, ADG, starter DMI, total DMI, FE, diarrhea rate, withers height, body length, heart girth, abdominal girth) were analyzed via a linear mixed model (LMM) with repeated measures, incorporating fixed effects of treatment, time, gender, and their interactions. Treatment group means were compared using one-way ANOVA after confirming normality via the Shapiro-Wilk test, followed by Duncan’s multiple range test for post-hoc pairwise comparisons. Diarrhea rates were analyzed using the Kruskal–Wallis test. Serum biochemical parameters were analyzed using general linear model (GLM) ANOVA, adjusting for gender and body weight as covariates to control for potential baseline variations.

## 3. Results

### 3.1. Growth Performance

Initial body weight (BW) of calves was not affected by treatment (*p* = 0.397). The overall BW in the M-MRF group was significantly higher than that in the control group by 4.9% (*p* < 0.001). Males exhibited significantly higher overall body weight (*p* < 0.001) and trended toward greater average daily gain (*p* = 0.068) compared to females. Calves in the L-MRF and M-MRF groups had significantly lower overall diarrhea rates (*p* = 0.046). All performance parameters were influenced by the experimental week. However, no significant differences in ADG, intake, FE, or diarrhea rate were detected among the control and MRF groups (*p* > 0.05) (Table 2).

Regarding other body measurement indices, the overall body length in the MRF groups was significantly greater than that in the control group, particularly in the L-MRF and M-MRF groups (*p* < 0.001). However, there were no significant effects on overall body height, heart girth, or abdominal girth (*p* > 0.05). Gender had significant effects on overall withers height, body length, heart girth, and abdominal girth (*p* < 0.001) (Table 3). 

### 3.2. Blood Parameters

At day 14, IL-12, GH, and IgG levels were significantly higher with MRF supplementation compared to the CON group (*p* < 0.05), while IL-2 level was only higher in the M-MRF group (*p* < 0.05). IL-6 and TNF-α levels were significantly lower in the MRF groups (*p* < 0.05).

**Figure 1:**
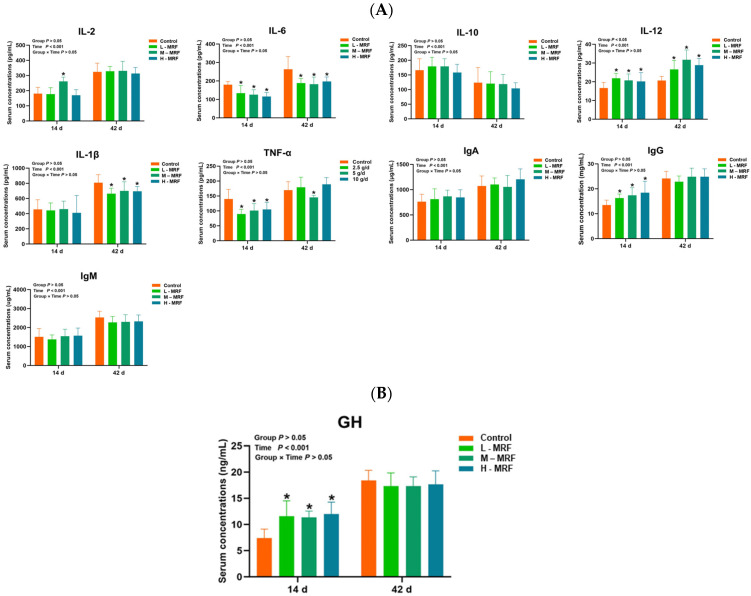
Effects of MRF on serum factors in dairy calves (*n* = 10). (**A**) Serum inflammatory factors; (**B**) GH (growth hormone). * *p* < 0.05. IL-2, Interleukin-2; IL-6, Interleukin-6; IL-10, Interleukin-10; IL-12, Interleukin-12; IL-1β, Interleukin-1β; TNF-α, Tumor Necrosis Factor-α; IgA, immunoglobulin A; IgG, immunoglobulin G; IgM, immunoglobulin M; GH, growth hormone. L-MRF = 2.5 g/d; M-MRF = 5 g/d; H-MRF = 10 g/d.

At day 42, IL-6 and IL-1β levels were significantly lower with MRF supplementation, while TNF-α level was only lower in the M-MRF group compared to the CON group (*p* < 0.05). Other parameters in the MRF-treated groups did not differ significantly from the control group (*p* > 0.05), as indicated in Figure 1.

### 3.3. Organ Index and GIT Morphometrics

**Figure 2:**
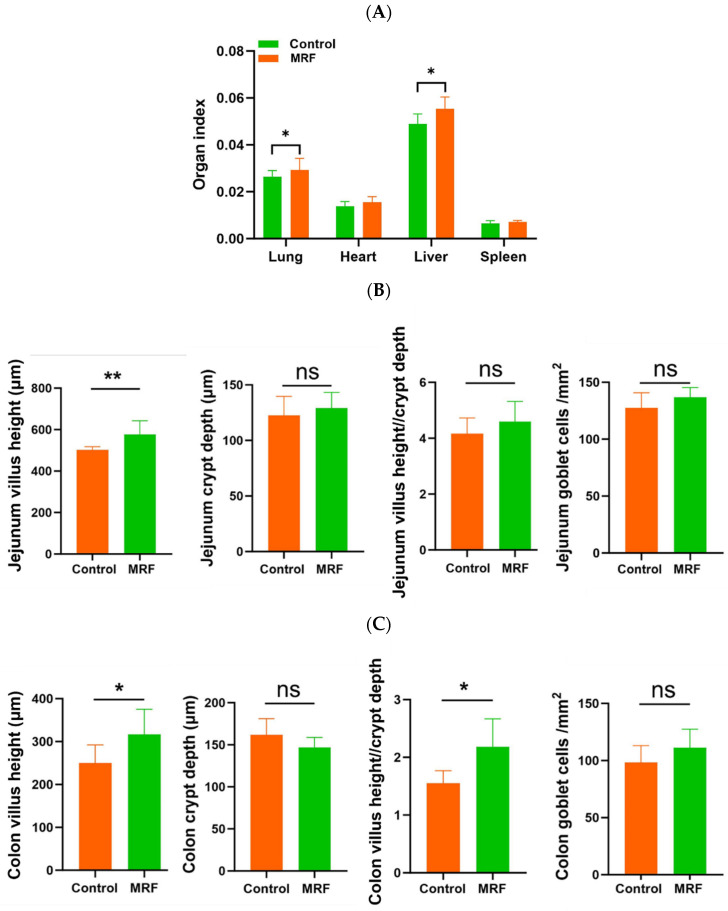

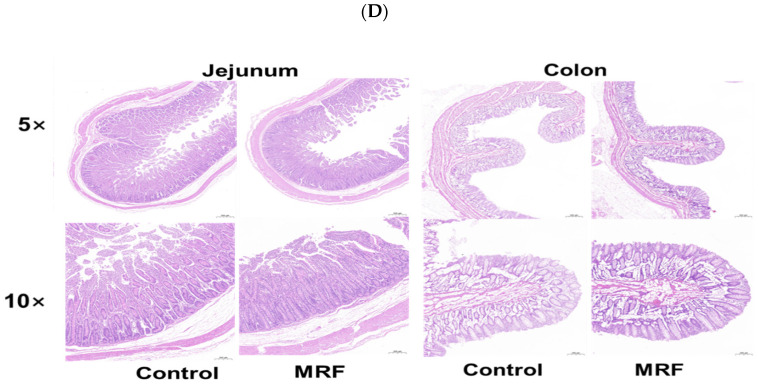
Effects of MRF on intestinal morphology in dairy calves (*n* = 6). (**A**) organ index; (**B**) villus height, crypt depth, villus height/crypt depth, and goblet cell number in the jejunum; (**C**) villus height, crypt depth, villus height/crypt depth, and goblet cell number in the colon; (**D**) intestinal morphology. MRF = 5 g/d. ** represents *p* < 0.01; * represents *p* < 0.05; ns represents *p* > 0.05.

The results showed that compared with the control group, the lung and liver indices of calves in the MRF group were significantly higher (*p* < 0.05). However, there were no significant differences in the heart and spleen indices compared with the control group (*p* > 0.05) (Figure 2A).

The intestinal morphology and goblet cell number were evaluated (Figure 2B,D). The MRF group had greater jejunum villus height compared to the CON group (*p* < 0.01). The calves in the MRF group showed a greater villus height and ratio of villus height to crypt depth in the colon than the CON group (*p* < 0.05). However, no differences in goblet cells were observed between the CON and MRF groups (*p* > 0.05).

### 3.4. Intestinal Biochemical and Immunological Parameters

**Figure 3:**
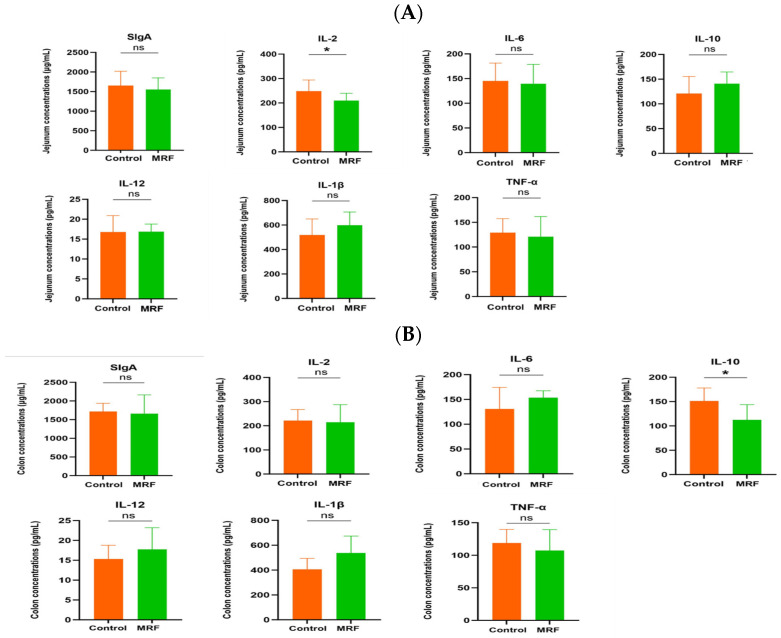
Effects of MRF on the jejunum and colon of dairy calves (*n* = 6). (**A**) Jejunum and (**B**) colon inflammatory factors. * *p* < 0.05. sIgA, secretory immunoglobulin A; IL-2, Interleukin-2; IL-6, Interleukin-6; IL-10, Interleukin-10; IL-12, Interleukin-12; IL-1β, Inter-leukin-1β; TNF-α, Tumor Necrosis Factor-α. MRF = 5 g/d. ns represents *p* > 0.05.

M-MRF fed calves had a lower IL-2 level in the jejunum (*p* < 0.05) and IL-10 level in the colon compared with the control group (*p* < 0.05). No statistically significant differences were observed in other measured parameters between the two groups, as shown in Figure 3.

### 3.5. SCFA Concentrations in the Jejunum and Colon

In the calves’ jejunum, the MRF group exhibited a significantly higher acetate level compared to the control group (*p* = 0.034). However, no significant differences were noted in the levels of other SCFAs. The total SCFA content in the MRF group trended upwards relative to the control group (*p* = 0.051). In terms of percentage composition, the control group had significantly higher proportions of isobutyrate (*p* = 0.005) and isovalerate (*p* = 0.011) than the MRF group (Table 4).

In the calves’ colons, the butyrate level in the MRF group was notably higher than that in the control group (*p* < 0.05). For other individual SCFAs and their respective proportions, no significant differences were observed between the two groups (Table 4).

### 3.6. Correlation Analysis Between SCFAs, Growth Performance and Inflammatory Factors

**Figure 4:**
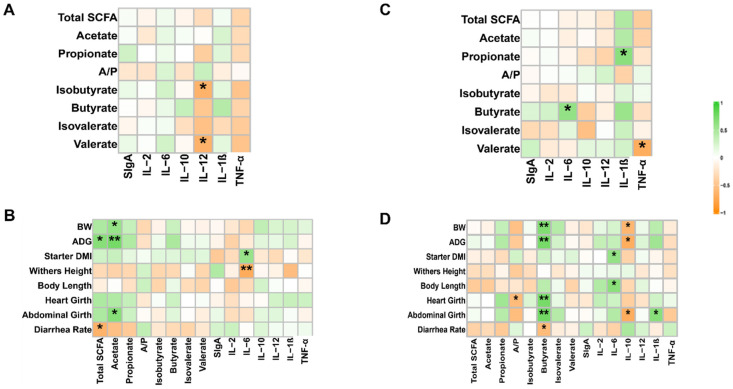
Correlation between phenotypes, SCFAs, and inflammatory factors (*n* = 6). (**A**) Correlation of jejunum SCFAs with inflammatory factors. Spearman’s correlation coefficients were used to evaluate associations. * *p* < 0.05. (**B**) Correlation of phenotypes with jejunum SCFAs and inflammatory factors. Spearman’s correlation coefficients were used to evaluate associations. * *p* < 0.05; ** *p* < 0.01. (**C**) Correlation of colon SCFAs with inflammatory factors. Spearman’s correlation coefficients were used to evaluate associations. * *p* < 0.05. (**D**) Correlation of phenotypes with colon SCFAs and inflammatory factors. Spearman’s correlation coefficients were used to evaluate associations. * *p* < 0.05, ** *p* < 0.01. Total SCFA, total short-chain fatty acids; A/P, the ratio of acetate/propionate; sIgA, secretory immunoglobulin A; IL-2, Interleukin-2; IL-6, Interleukin-6; IL-10, Interleukin-10; IL-12, Interleukin-12; IL-1β, Inter-leukin-1β; TNF-α, Tumor Necrosis Factor-α.

In the jejunum, total SCFA showed a significant positive correlation with ADG and a negative correlation with diarrhea rate. Acetate was significantly positively correlated with BW, ADG, and abdominal girth. IL-6 was negatively correlated with withers height and positively correlated with starter intake. IL-12 was negatively correlated with isobutyrate and valerate (Figure 4A,B).

In the colon, butyrate was significantly positively correlated with BW, ADG, heart girth, and abdominal girth and was negatively correlated with diarrhea rate. IL-6 was positively correlated with starter intake and body length. IL-10 was negatively correlated with BW, ADG, and abdominal girth. IL-6 was positively correlated with butyrate, IL-1β was positively correlated with propionate, and TNF-α was negatively correlated with valerate (Figure 4C,D).

## 4. Discussion

The raising of young animals holds significant importance as it is pivotal for ensuring their lifelong productive performance [23,24,25]. In the early developmental stage, the digestive tract of young animals, such as calves, experiences crucial changes. During this period, appropriate nutritional regulation emerges as a key factor in optimizing their production potential. In the context of nutritional interventions, research has shown that MOS supplementation in piglets can enhance gut health and reduce diarrhea [15]. This finding implies a potential positive impact on the health of pre-weaning calves, highlighting MOS as a valuable nutritional option in calf rearing. Consistent with the results obtained by another research study [10], the MRF supplementation, especially the M-MRF group, exhibited higher BW, body length, and starter intake compared with the control group. However, Ann was unaffected, a finding that aligns with a previous study [26]. It is worth noting that some studies have reported that adding MOS to the starter or milk replacer did not improve the growth performance of calves [27]. The divergence in these results may be attributed to weaning stress on day 42 overshadowing the effects of MOS. Understanding the specific benefits to the production performance of calves fed with MRF and its mechanism of action is crucial, only in this way can we ensure the use of appropriate supplements in the correct form at the right time to promote calf growth and productivity.

Diarrhea is a common and important problem among young animals, and it can lead to stunted growth and developmental delays. Research indicates that MOS binds to mannose-specific lectin receptors on the outer membranes of Escherichia coli and Salmonella enterica, thereby blocking their adhesion to the gut wall [28]. This prevents colonization and proliferation to pathogenic levels, ultimately enhancing immune function and reducing diarrhea rates in piglets and calves [15,29]. In this study, the L-MRF and M-MRF groups had a lower diarrhea rate, likely due to lower pathogenic bacteria following MRF intervention [30]. Moreover, in this study MRF-supplemented animals had lower serum levels of IL-6, IL-1β, TNF-α and higher IgG level at d 14, aligning with prior observations in piglets [15,31]. IL-6, IL-1β, and TNF-α, as crucial proinflammatory cytokines, play pivotal roles in orchestrating immune responses and modulating inflammation [32,33] When pathogens attach to the intestinal mucosa, they can trigger a series of immune responses, leading to the production and release of inflammatory cytokines such as TNF-α, IL-1β, and IL-6. However, with the addition of MOS, this process was disrupted [34]. IL-1β not only initiates and amplifies inflammatory processes, but it also induces the secretion of IL-6 and TNF-α, with its levels being linked to the degree of intestinal inflammation [35,36]. The lower levels of blood inflammatory factors and higher levels of immune markers in the MRF-supplemented groups suggest that MRF may mitigate systemic inflammation in pre-weaning calves.

The intestinal morphological index is frequently regarded as a key measure of the body’s capacity to digest and absorb nutrients. The villus height is a crucial structure of the small intestine that is primarily involved in nutrient absorption [37]. Functional oligosaccharides have been found to enhance dairy goat kid performance by boosting nutrient absorption through improved intestinal morphology [10,38]. Previous work also showed greater villus height with MOS supplementation in pre-weaning piglets and poultry [14,39]. In our study, MRF-fed calves had greater jejunum and colon villus height compared to the CON group, suggesting that the intestine’s absorption capacity may be greater with MRF.

The lower jejunal IL-2 and colonic IL-10 levels implied a targeted immunomodulatory effect of MRF. This may be due to mitigation of harmful bacteria by MRF and consequential beneficial shifts in the intestinal microbial environment. MRF has been shown to bind to specific mannose lectin receptors on pathogenic bacteria, effectively reducing their presence in the intestine, and it may curb the adverse impact of harmful bacteria on the intestinal microbiota by doing so [39]. A healthier microbial environment lowers the risk of immune over-activation [40]. IL-2 is crucial for T-lymphocyte activation and proliferation, and lower levels in the jejunum may suggest that MRF is regulating local immune activation in this region, potentially preventing over-activation [41]. This helps maintain immune homeostasis, ensuring that the immune response is appropriately regulated and does not cause unnecessary inflammation. IL-10, a powerful suppressor of Th1 cytokines, restrains the production of pro-inflammatory cytokines like TNF-α and IL-6 and is crucial for sustaining immune homeostasis and averting immune-related inflammatory disorders triggered by pathogens [42,43]. In the colon, the decrease in IL-10 level might seem counterintuitive at first. To understand this phenomenon, it is essential to consider the unique characteristics of the intestinal environment. Different regions of the intestine have distinct microbiota compositions and immune cell distributions [44]. It is hypothesized that upon application of MRF, it may preferentially target and inhibit harmful bacteria in the colon [28]. As a result, the threat of inflammation posed by these pathogens is reduced. With a lower pathogenic load, the body may no longer require high levels of IL-10 to counteract excessive inflammation, thus leading to a decrease in its levels.

SCFAs are produced by the fermentation of dietary fiber by gut microbiota and have been shown to exert substantial influence on host health, including providing energy for the intestinal epithelium, modulating immune function, and maintaining intestine health [45,46,47]. The jejunum of calves in the MRF group had significantly higher acetate levels compared to the control group, aligning with the higher acetate levels in the cecum of poultry fed MOS [48]. The greater acetate might be related to MRF-induced shifts in the jejunal microbiota [49]. Although the levels of other SCFAs did not differ significantly, the upward trend in total SCFAs in the MRF group indicates an overall difference in SCFA production.

In the colon of calves, the butyrate level in the MRF group was notably higher than that in the control group. Butyrate, a key short-chain fatty acid (SCFA) abundant in the colon, plays a pivotal role in calf colon health during development [50]. Research has demonstrated that butyrate is crucial for maintaining colon health, as it can significantly promote intestinal development and improve gut health. It serves as the main energy source for colonocytes and influences various cellular functions related to colonic health [51]. Adequate butyrate is essential for maintaining colonic epithelial integrity. Insufficient butyrate makes colonocytes more likely to undergo apoptosis, disrupting the intestinal barrier and increasing permeability [52,53]. Moreover, butyrate has potent anti-inflammatory properties, mainly achieved by inhibiting the NF-κB signaling pathway. In normal circumstances, NF-κB, a transcription factor, is sequestered in the cytoplasm by its inhibitor IκBα. However, in response to inflammatory stimuli, IκBα is phosphorylated, ubiquitinated, and degraded, allowing NF-κB to translocate to the nucleus and initiate the transcription of pro-inflammatory cytokines such as TNF-α, IL-1β, and IL-6. Butyrate reduces the production of pro-inflammatory cytokines by inhibiting NF-κB activation and IκBα degradation [54]. The observed taller villi in the MRF-treated calves are likely a result of the combined effects of enhanced energy supply and the anti-inflammatory environment created by butyrate. Given these known functions of butyrate and the observed higher butyrate levels in the MRF-treated calves, it is reasonable to suggest that the MRF treatment may have a positive impact on colon health.

The positive correlation between SCFAs—primarily acetate and butyrate—and phenotypic indicators in both the jejunum and colon aligns with their well-known functions in promoting intestine epithelial cell growth and function [55,56,57,58]. In this study, dietary supplementation with MRF resulted in significantly higher acetate concentration in the jejunum and butyrate concentration in the colon. The negative correlation between SCFAs and diarrhea rate further supports their role in maintaining gut health, underscoring the beneficial impact of MRF on gut health through SCFAs regulation. The relationship between cytokines and phenotypic indicators is more complex. IL-6, which is a proinflammatory cytokine, was positively correlated with starter intake in both the jejunum and colon. However, it was negatively correlated with withers height in the jejunum. This suggests that IL-6 may have different effects on different aspects of growth [59]. The negative correlation between IL-12 and SCFAs in the jejunum may suggest that during pathogenic infections, elevated IL-12, which is released as part of the immune response to combat pathogens, disrupts the normal production of SCFAs by gut microbiota [60]. The negative correlation between IL-10 and growth parameters in the colon is somewhat unexpected, as IL-10 is generally considered an anti-inflammatory cytokine that promotes tissue repair and regeneration [61]. This phenomenon may occur because the balance of cytokines, including IL-10, in the colon is highly susceptible to changes in the microbiota. When the microbiota is disrupted—an event likely associated with the negative correlation between IL-10 levels and growth parameters—it can undermine essential colonic functions required for normal growth [60]. The positive correlation between IL-6 and butyrate as well as IL-1β and propionate and the negative correlation between TNF-α and valerate suggest that cytokines and SCFAs may interact with each other to modulate gut function [62]. Given MRF’s role in enhancing acetate and butyrate levels, it may also indirectly influence the cytokine-SCFA interplay, beneficially contributing to gut function and calf health.

Despite its valuable findings, this study has limitations. First, as climates and environments vary greatly across regions, the results may not apply to other areas or commercial farms. Second, the study only examined short-term effects of MRF during the pre-weaning period; long-term impacts on post-weaning growth and health remain unknown. Finally, while multiple parameters were investigated, the molecular mechanisms of MRF’s effects on calves, including relevant signaling pathways, still need further exploration.

## 5. Conclusions

The study highlights the benefits associated with MRF supplementation on the growth performance and health of pre-weaning calves. MRF supported intestinal morphology and growth while allowing lower diarrhea incidence. The higher jejunal acetate and colonic butyrate in MRF-supplemented calves further validates its positive impact on gut health. These findings establish 5 g/day MRF as the optimal early-life supplementation strategy for optimizing the productive performance and health of young animals. Future research should explore MRF’s effects on weaning stress and lactation performance in dairy heifers as well as its potential as a sustainable alternative to antibiotics in calf rearing.”

## Figures and Tables

**Table 1:** The chemical composition of starter used in the control and supplementation diets.

Item	Starter ^1^
Dry Matter	88.3
Crude Protein, %DM	20.45
Ether Extract, %DM	3.35
Starch, %DM	21.74
NDF, %DM	20.16
ADF, %DM	9.86
Ash, %DM	7.63
Ca, %DM	0.85
P, %DM	0.57
Mg, %DM	0.21

^1^ Provided by Cofco Feed Group Co., Ltd. (Yinchuan China). and containing the following main ingredients: corn, soybean meal, corn gluten feed, and wheat bran. DM expressed as % of as-fed basis.

**Table 2:** Effects of MRF on the growth performance, starter intake, and diarrhea rate of dairy calves (*n* = 15/treatment, 7 male and 8 female) ^1^.

	Treatment				SEM	Gender		SEM	*p*-Value						
Item	Control	L-MRF	M-MRF	H-MRF		Male	Female		T	W	G	T × W	T × G	W × G	T × W × G
BW, kg															
d 7	47.96 ^b^	48.23 ^ab^	50.30 ^a^	47.16 ^b^	0.537	51.35	45.84	0.445	0.038		<0.001				
d 14	53.36	53.40	55.60	53.10	0.558	56.76	51.32	0.525	0.158		<0.001				
d 21	61.00	60.57	63.70	60.77	0.692	65.00	58.45	0.753	0.134		<0.001				
d 28	67.63 ^b^	68.17 ^b^	71.43 ^a^	67.73 ^b^	0.676	72.57	65.39	0.658	0.014		<0.001				
d 42	79.93	80.63	84.06	80.43	0.762	85.10	77.90	0.836	0.056		<0.001				
Overall	61.98 ^b^	62.20 ^b^	65.02 ^a^	61.84 ^b^	0.602	66.16	59.78	0.425	0.027	<0.001	<0.001	0.466	0.134	0.034	0.387
ADG, kg/d															
d 1–7	0.82	0.83	0.91	0.93	0.035	0.92	0.84	0.043	0.480		0.221				
d 8–14	0.77	0.74	0.76	0.85	0.037	0.77	0.78	0.517	0.774		0.889				
d 15–21	1.09	1.02	1.16	1.09	0.057	1.17	1.01	0.078	0.902		0.160				
d 22–28	0.95	1.08	1.10	0.99	0.040	1.08	0.99	0.055	0.452		0.254				
d 29–42	0.88	0.89	0.90	0.91	0.020	0.90	0.89	0.028	0.954		0.968				
Overall	0.90	0.91	0.97	0.96	0.050	0.97	0.90	0.035	0.186	0.003	0.068	0.495	0.095	0.108	0.087
Starter Intake, g/d															
d 1–7	4.49	6.89	5.96	3.13	3.345	5.77	4.54	0.837	0.653		0.607				
d 8–14	14.75	15.17	15.24	13.04	7.376	19.56	10.17	2.200	0.989		0.076				
d 15–21	11.73	18.53	17.33	16.85	5.742	16.09	16.13	2.714	0.634		0.993				
d 22–28	19.40	34.80	31.14	16.35	11.291	23.31	27.27	5.688	0.310		0.628				
d 29–35	41.75	62.16	55.52	41.64	15.630	39.79	59.44	5.964	0.425		0.078				
d 36–42	62.10	101.19	68.92	78.10	23.257	60.26	92.73	8.598	0.337		0.053				
Overall	25.71	39.79	32.35	28.19	5.286	27.47	49.97	3.738	0.252	<0.001	0.166	0.348	0.470	0.016	0.727
Total DMI, kg/d															
d 1–7	1.02	1.03	1.03	1.02	0.001	1.02	1.02	0.001	0.480		0.423				
d 8–14	1.22	1.22	1.22	1.22	0.002	1.22	1.21	0.002	0.969		0.074				
d 15–21	1.39	1.40	1.40	1.40	0.002	1.39	1.39	0.003	0.474		0.801				
d 22–28	1.41	1.44	1.43	1.42	0.004	1.42	1.42	0.005	0.264		0.655				
d 29–42	1.46	1.50	1.47	1.48	0.008	1.49	1.49	0.008	0.324		0.045				
Overall	1.30	1.32	1.31	1.31	0.006	1.31	1.31	0.004	0.188	<0.001	0.305	0.249	0.552	0.013	0.660
FE															
d 1–7	0.81	0.81	0.89	0.91	0.342	0.90	0.82	0.042	0.464		0.221				
d 8–14	0.63	0.60	0.62	0.69	0.305	0.63	0.64	0.042	0.768		0.832				
d 15–21	0.78	0.73	0.83	0.78	0.408	0.84	0.73	0.055	0.890		0.156				
d 22–28	0.66	0.76	0.77	0.70	0.028	0.76	0.69	0.039	0.528		0.229				
d 29–42	0.60	0.59	0.62	0.61	0.134	0.61	0.60	0.017	0.912		0.637				
Overall	0.70	0.70	0.74	0.74	0.038	0.75	0.70	0.027	0.147	<0.001	0.010	0.470	0.097	0.732	0.052
Diarrhea Rate, %															
d 1–7	25.72 ^ab^	25.72 ^ab^	11.43 ^b^	35.24 ^a^	8.275	19.39	29.01	3.833	0.021		0.141				
d 8–14	8.57	5.71	6.66	12.38	5.518	8.16	8.48	2.692	0.228		0.950				
d 15–21	19.04	9.52	16.19	24.76	6.514	14.79	19.64	3.072	0.222		0.395				
d 22–28	15.24	13.33	8.57	11.42	5.302	14.28	10.26	2.420	0.530		0.247				
d 29–35	5.71	5.71	0.95	2.85	3.158	4.59	3.12	1.239	0.374		0.904				
d 36–42	2.86	3.81	5.71	1.91	2.846	3.57	3.57	1.399	0.645		0.848				
Overall	12.86 ^a^	10.64 ^ab^	8.25 ^b^	14.76 ^a^	2.293	10.80	12.35	1.621	0.046	<0.001	0.339	0.009	0.259	0.049	0.731

^1,a,b^ means within a row with different superscripts differ significantly (*p* < 0.05). BW, body weight; ADG, average daily gain. Starter intake was recorded and calculated on as-fed basis. Total DMI, total dry matter intake, was calculated as the sum of the DMI of milk and starter. FE, feed efficiency = ADG/Total DMI; T, treatment; W, week; G, gender. L-MRF = 2.5 g/d; M-MRF = 5 g/d; H-MRF = 10 g/d.

**Table 3:** Effects of MRF on body measurement indexes of dairy calves (*n* = 15/treatment, 7 male and 8 female) ^1^.

	Treatment				SEM	Gender		SEM	*p*-Value						
Item	Control	L-MRF	M-MRF	H-MRF		Male	Female		T	W	G	T × W	T × G	W × G	T × W × G
Withers Height, cm															
d 7	81.40	82.73	83.40	82.40	0.363	85.53	81.56	0.408	0.229		0.005				
d 14	83.40	84.27	85.13	84.67	0.323	85.64	83.25	0.339	0.170		<0.001				
d 21	84.27	83.87	85.07	84.33	0.301	85.10	83.75	0.406	0.531		0.026				
d 28	85.80	87.53	87.07	86.60	0.341	87.75	85.87	0.432	0.264		0.006				
d 42	88.47	88.53	88.40	89.93	0.418	90.28	87.56	0.533	0.475		<0.001				
Overall	84.67 ^b^	85.38 ^ab^	85.81 ^a^	85.59 ^a^	0.416	86.46	84.40	0.294	0.365	<0.001	<0.001	0.231	0.489	0.603	0.790
Body Length, cm															
d 7	70.60	71.73	72.13	70.73	0.301	72.00	70.69	0.371	0.178		0.029				
d 14	73.73 ^c^	75.27 ^b^	76.73 ^a^	74.80 ^c^	0.276	76.03	74.34	0.331	<0.001		<0.001				
d 21	74.60 ^ab^	75.33 ^ab^	76.00 ^a^	73.53 ^b^	0.363	75.68	74.16	0.449	0.049		0.025				
d 28	78.93 ^b^	80.53 ^ab^	82.40 ^a^	80.40 ^b^	0.382	81.71	79.56	0.448	0.008		0.002				
d 42	85.00	85.87	86.67	85.27	0.465	87.11	84.47	0.527	0.538		0.004				
Overall	76.57 ^c^	77.75 ^b^	78.79 ^a^	76.95 ^c^	0.421	78.51	76.64	0.298	<0.001	<0.001	<0.001	0.968	0.637	0.120	0.906
Heart Girth, cm															
d 7	88.93	89.47	89.33	89.00	0.339	90.32	88.19	0.343	0.886		0.001				
d 14	91.07	91.33	90.67	90.07	0.319	92.07	89.66	0.369	0.437		<0.001				
d 21	94.60	93.33	94.20	93.93	0.352	95.21	92.97	0.402	0.627		0.001				
d 28	94.53	95.73	96.87	95.27	0.372	97.07	94.31	0.398	0.081		<0.001				
d 42	101.73	102.40	102.20	101.73	0.488	103.53	100.68	0.55	0.937		0.003				
Overall	94.17	94.45	94.65	94.00	0.440	95.64	93.16	0.311	0.759	<0.001	<0.001	0.854	0.293	0.407	0.089
Abdominal Girth, cm															
d 7	95.20	96.13	97.60	95.67	0.450	98.07	94.47	0.491	0.161		<0.001				
d 14	98.60	97.80	98.73	98.47	0.436	99.64	97.31	0.447	0.869		0.009				
d 21	101.33	100.87	102.00	102.13	0.576	103.43	99.97	0.653	0.850		0.003				
d 28	101.00	102.73	102.93	101.47	0.531	103.96	100.34	0.673	0.434		<0.001				
d 42	107.60	109.60	109.00	110.93	0.637	110.93	107.84	0.857	0.245		0.014				
Overall	100.75	101.43	102.05	101.73	0.636	103.21	99.99	0.450	0.530	<0.001	<0.001	0.190	0.729	0.923	0.816

^1,a,b,c^ means within a row with different superscripts differ significantly (*p* < 0.05). T, treatment; W, week; G, gender. L-MRF = 2.5 g/d; M-MRF = 5 g/d; H-MRF = 10 g/d.

**Table 4:** Concentration of SCFAs of calves fed with and without MRF ^1^.

	Treatment			
	Control	MRF	SEM	*p*-Value
Jejunum				
Concentration				
Acetate (mmol/L)	5.37	19.74	5.087	0.034
Propionate (mmol/L)	2.04	5.11	1.775	0.120
Isobutyrate (mmol/L)	0.21	0.33	0.187	0.526
Butyrate (mmol/L)	0.84	1.88	0.712	0.177
Isovalerate (mmol/L)	0.80	0.82	0.261	0.926
Valerate (mmol/L)	0.17	0.36	0.280	0.524
A/P ^2^	5.06	2.87	1.249	0.443
Total SCFA ^3^	9.45	28.26	7.782	0.051
Proportion				
Acetate, %	63.66	70.36	5.741	0.282
Propionate, %	16.75	17.99	4.034	0.768
Isobutyrate, %	2.07	0.91	0.322	0.005
Butyrate, %	6.87	6.72	2.416	0.951
Isovalerate, %	9.50	3.30	1.998	0.011
Valerate, %	1.12	0.70	0.814	0.623
Colon				
Concentration				
Acetate (mmol/L)	60.75	67.19	13.491	0.643
Propionate (mmol/L)	10.48	13.61	2.787	0.288
Isobutyrate (mmol/L)	0.42	0.41	0.153	0.933
Butyrate (mmol/L)	4.43	6.37	0.658	0.029
Isovalerate (mmol/L)	0.91	1.33	0.300	0.184
Valerate (mmol/L)	0.23	0.71	0.379	0.262
A/P ^2^	6.02	5.16	0.887	0.352
Total SCFA ^3^	77.23	89.64	16.242	0.463
Proportion				
Acetate, %	77.76	74.68	2.758	0.290
Propionate, %	13.62	15.37	2.040	0.412
Isobutyrate, %	0.62	0.45	0.190	0.379
Butyrate, %	6.32	7.31	0.999	0.344
Isovalerate, %	1.32	1.56	0.377	0.536
Valerate, %	0.34	0.62	0.302	0.381

^1^*n* = 6 for all variables in each treatment group of each indicator. MRF = 5 g/d. ^2^ A/P, the ratio of acetate/propionate. ^3^ Total SCFA, total short-chain fatty acids.

## Data Availability

The original contributions presented in this study are included in the article. Further inquiries can be directed to the corresponding author.

## Abbreviations

The following abbreviations are used in this manuscript:

MRF	Mannan-rich fraction
MOS	Mannan oligosaccharides
SCFA	Short-chain fatty acid
BW	Body weight
ADG	Average daily gain
IL-2	Interleukin-2
IL-6	Interleukin-6
IL-10	Interleukin-10
IL-12	Interleukin-12
IL-1β Interleukin-1β	Interleukin-1β
TNF-α Tumor Necrosis Factor-α	Tumor Necrosis Factor-α
GH Growth Hormone	Growth Hormone
IgA Immunoglobulin A	Immunoglobulin A
IgG Immunoglobulin G	Immunoglobulin G
IgM Immunoglobulin M	Immunoglobulin M
sIgA secretory immunoglobulin A	Secretory immunoglobulin A
